# Editorial: Adrenal insufficiency: Diagnostic approaches, treatments and outcomes

**DOI:** 10.3389/fendo.2023.1180264

**Published:** 2023-04-12

**Authors:** A. Pazderska, C. Malchoff, O. Ragnarsson, E. Valassi

**Affiliations:** ^1^ Department of Endocrinology, St. James’s Hospital, Dublin, Ireland; ^2^ School of Medicine, Trinity College Dublin, Dublin, Ireland; ^3^ Department of Medicine, University of Connecticut Health Center, Farmington, CT, United States; ^4^ Institute of Medicine, Sahlgrenska Academy, University of Gothenburg, Göteborg, Sweden; ^5^ Department of Endocrinology, Sahlgrenska University Hospital, Göteborg, Sweden; ^6^ Wallenberg Center for Molecular and Translational Medicine, University of Gothenburg, Göteborg, Sweden; ^7^ Endocrinology and Nutrition Department, Germans Trias i Pujol Hospital and Research Institute, Badalona, Spain; ^8^ Centro de Investigación Biomédica en Red de Enfermedades Raras (CIBERER), Universitat Internacional de Catalunia (UIC), Barcelona, Spain

**Keywords:** adrenal crisis, adrenal insufficiency, glucocorticoids, diagnosis of adrenal insufficiency, cortisol, metyrapone test

The diagnosis and treatment of adrenal insufficiency (AI) remains a challenge for both patients and clinicians. Long term studies reveal that patients with adrenal insufficiency have increased morbidity, mortality and impaired quality of life (1). Possible contributors are failure of the currently-available glucocorticoid (GC) replacement formulations to mimic the physiological diurnal secretion of cortisol, high incidence of infection, and suboptimal patient education, amongst others. It is therefore gratifying that a recent issue of Frontiers in Endocrinology is dedicated to novel research on adrenal insufficiency.

GCs are widely used for treatment of various conditions and are the most common cause of adrenal insufficiency (2). In this issue, Einarsdottir et al. report findings from a large population-based study investigating all-cause and disease-specific mortality amongst oral GC users, defined as patients receiving ≥5 mg prednisolone, or equivalent dose of other GCs, for three weeks or longer. GC users had increased all-cause mortality (HR adjusted for age, sex and comorbidities (95%CI) 2.08 (2.04-2.13), p<0.0001) compared to controls. The excess deaths were mainly due to pulmonary embolism, pneumonia and sepsis- mirroring complications observed in endogenous hypercortisolism. Whether the high mortality is caused by GC treatment, or merely reflects the severity of the underlying medical condition, remains unknown.

Patients with AI are at increased risk of infections, and hospitalizations due to infections (3, 4). During the COVID-19 pandemic concerns were raised about the susceptibility to, and potentially worse outcomes in SARS-CoV-2 infections, amongst AI individuals. Yedinak et al. conducted a 27-item survey amongst AI patients, recruited world-wide *via* social media, websites and advocacy groups, to determine self-reported incidence of COVID-19 infection. Overall, 3.1% of 1291 respondents with AI tested positive for COVID-19, compared to 1.3% global cumulative incidence for the same time-period. 22.5% of COVID-19 positive AI individuals required hospitalization with a risk ratio that was 24-fold higher than in the global population. While the data is striking, like many studies on incidence of COVID infections in specific populations, self-selection bias, inability to verify diagnosis and recall bias, may have contributed.

The diagnosis of AI is based on demonstrating suboptimal cortisol response to 250 µg of synthetic ACTH. Some argue that this dose represents a supraphysiological stimulus and may lead to false-negative results, particularly in secondary AI. The insulin tolerance test, once considered a “gold standard” for the diagnosis of secondary AI, is utilized less commonly nowadays as it is labor-intense and carries risks associated with hypoglycaemia. The metyrapone test is an alternative to diagnose secondary AI. However, the use of this test is limited by access to 11-deoxycortisol measurements. Papierska et al. investigated if a single measurement of ACTH, instead of 11-deoxycortisol, during the metyrapone test, can provide acceptable diagnostic accuracy. The optimal cut-off of ACTH during the metyrapone test was estimated at 147 ng/l with a sensitivity of 71% and specificity of 84%. However, even with an ACTH diagnostic threshold of >200 ng/L, false negative results were present in 20% of secondary AI patients, highlighting that a single ACTH measurement cannot replace 11-deoxycortisol measurements.

Ali et al. report an eight-year-old boy with isolated primary glucocorticoid deficiency with hypoplastic adrenal glands possibly due to combined digenic, tri-allelic inheritance of two *STAR* (steroidogenic acute regulator protein) mutations and a *CYP11A1* (encoding the P450 side chain cleavage enzyme) variant, which is the first description of digenic, tri-allelic primary AI.

Cognitive dysfunction is common in patients with hypercortisolism which may persist after reversal of the cortisol excess. Pupier et al. evaluated patients younger than 60 years of age in remission following surgical treatment for Cushing disease (excluding relevant comorbidities and psychotropic use) and show that memory impairment is not present at long-term follow-up. However, the patients displayed impaired quality-of-life, the intensity of which was proportional to the duration of active hypercortisolism prior to the cure, underscoring the importance of early diagnosis and treatment.

Adrenal crisis affects a substantial proportion of patients with AI, with an incidence of approximately 5-10 per 100-patient-years, with many suffering from recurrent episodes (5). However, little is known about the epidemiology of adrenal crises in adolescents and young adults who may be particularly vulnerable to this complication because of the challenges associated with transition to adulthood. Chrisp et al. analyzed all hospital admissions in Australia between 2000/1 to 2019/20 for AI, including adrenal crises, in 10 -24-year-olds. The authors showed that between 2000/1 and 2019/20 admission rates for adrenal crises increased, especially in young women aged 20-24 years where it increased from 3/million to 40/million. This concerning trend is unexplained and has not been reported previously.

To date, few risk factors for adrenal crises have been identified which limits application of preventive measures. Vulto et al. investigated a biological predisposition to adrenal crisis by studying cortisol pharmacokinetics and pharmacodynamics in patients with secondary AI. Patients with history of adrenal crises demonstrated differences in cortisol and cortisone excretion, as well as metabolomic profiles suggestive of reduced glucocorticoid sensitivity compared with those who did not experience adrenal crisis.

Current guidelines for treatment of AI advocate the use of lower doses of short-acting GCs, equivalent to a daily dose of 15-25 mg of hydrocortisone(HC) (6).Higher daily GC doses have been associated with cardiovascular morbidity and mortality and infections (7). Caetano et al. argue that the currently-recommended replacement doses may lead to overtreatment in at least some patients. They present a titration method (reducing GC dose by 5 mg of HC equivalent every 2-6 months) for determination of the individual’s optimal daily GC dose, based on empirical assessment of AI symptoms. Using this method, the mean daily HC dose equivalent achieved was 13.9 ± 6 mg (7.6 ± 3.4 mg/m^2^), approximating the daily cortisol production rate of 7 mg/m^2^, and significantly lower than the current recommendations.

Replacement GC, especially in excess doses, can affect bone quality in patients with AI (8). Zdrojowy-Wełna et al. evaluated densitometry parameters, and trabecular bone score in 29 patients with primary AI. There was no difference in T-score, Z-score, bone mineral density or trabecular bone score (TBS) in patients compared to controls. There was, however, a negative correlation between TBS and the duration of AI and age, as well as between densitometry parameters and 24-hour urinary cortisol, indicating that disease duration and higher HC doses may affect bone status negatively.

We hope that this Research Topic provides a valuable resource on the current knowledge regarding several aspects of clinically relevant topics associated with adrenal insufficiency, its pathophysiology, diagnosis and treatment.

## Author contributions

All authors listed have made a substantial, direct, and intellectual contribution to the work and approved it for publication.
